# Design of Novel Mechanically Resistant and Biodegradable Multichannel Platforms for the Treatment of Peripheral Nerve Injuries

**DOI:** 10.1021/acs.biomac.2c01498

**Published:** 2023-03-16

**Authors:** Caterina Valentino, Barbara Vigani, Gaia Zucca, Marco Ruggeri, Giorgio Marrubini, Cinzia Boselli, Antonia Icaro Cornaglia, Giuseppina Sandri, Silvia Rossi

**Affiliations:** † Department of Drug Sciences, University of Pavia, Viale Taramelli, 12, 27100 Pavia, Italy; ‡ Department of Public Health, Experimental, and Forensic Medicine, University of Pavia, Via Forlanini 2, 27100 Pavia, Italy

## Abstract

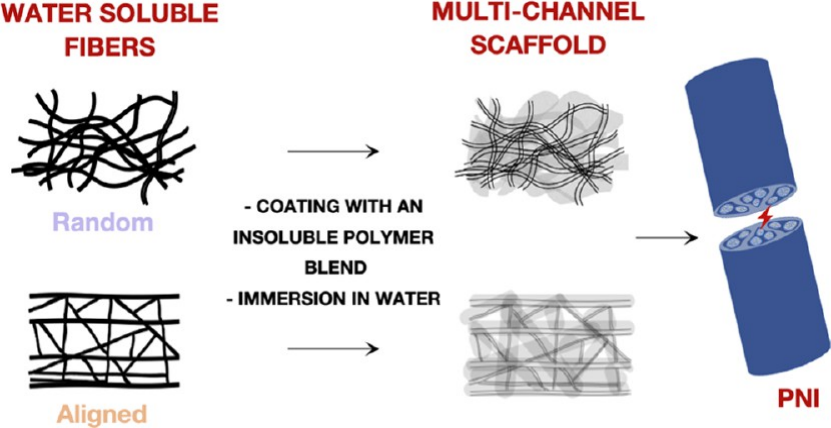

Peripheral nerve injury is one of the most debilitating pathologies that severely impair patients’ life. Although many efforts have been made to advance in the treatment of such a complex disorder, successful strategies to ensure full recovery are still scarce. The aim of the present work was to develop flexible and mechanically resistant platforms intended to act as a support and guide for neural cells during the regeneration process of peripheral nerve injury. For this purpose, poly(lactic-*co*-glycolic acid) (PLGA)/poly(d,l-lactic acid) (PDLLA)/poly(ethylene glycol) 400 (PEG)-multichannel-based scaffolds (MCs) were prepared through a multistep process involving electrospun microfibers coated with a polymer blend solution and used as a sacrificial mold. In particular, scaffolds characterized by random (MCR) and aligned (MCA) multichannel were obtained. A design of experiments approach (DoE) was employed to identify a scaffold-optimized composition. MCs were characterized for morphological and mechanical properties, suturability, degradability, cell colonization, and in vivo safety. A new biodegradable, biocompatible, and safe microscale multichannel scaffold was developed as the result of an easy multistep procedure.

## 1 Introduction

Peripheral nerve injuries (PNIs) are generally caused by traumatic events, such as vehicle accidents, gunshot, or sports, but the causes also include immune diseases and genetic factors.^1,2^

As for trauma mechanisms, they are mainly mechanical in nature (compression, traction, transection); less common mechanisms include friction, pressure, and finally, traumatic injury related to thermal or electrical insults or exposure to radiation.^1,3^

Despite PNI having a lower incidence when compared to other neuronal injuries such as spinal cord injury (SCI), it usually results in a severe impairment of patients’ quality of life due to the damage to motor and sensory functions.^4−6^ PNI, especially if trauma-related, involves a primary injury, which results directly from the trauma undergone, and a secondary injury, due to the vascular ischemic damage that usually follows. Concurrent damage to blood vessels is associated with the formation of a compressive hematoma, leading to nerve ischemia and exacerbation of the primary lesion. Unlike the central nervous system (CNS), the peripheral nervous system (PNS) has an intrinsic natural capability of regeneration after injury; nevertheless, regeneration is often incomplete, and consequently, the resulting motor recovery is scarce and the sensory function is not recovered.^7,8^

Up to now, an effective therapeutic strategy that can lead to a complete resolution of PNI is lacking.^8,9^ In fact, the prospects of complete sensory and motor recovery after injury are currently scarce; among the factors affecting a proper recovery there are the length of the gap, the time elapsed between injury and treatment, and the age of the patient.^10^

In addition to standard treatments such as neurorrhaphy and grafting (auto- or allografts), which bring with them numerous disadvantages,^11^ tissue-engineered grafts, called nerve guide/guidance conduits (NGCs), have been gaining increasingly more attraction in the last decades, representing nowadays a promising therapeutic strategy to promote the regeneration of nerve tissue lesions.^12^ Basically, the key concept for the employment of a NGC is the application of a hollow tube to bridge the proximal and distal nerve endings, supplying an aligned macroenvironment for nerve growth.^13,14^ For this purpose, numerous materials, both synthetic and natural, different designs and configurations, and various production techniques are reported in the literature, as meticulously described by Vijayavenkataraman^10^ and Wieringa and co-workers.^9^ In particular, it was proven that an electrospun fibrous NGC structure can be easily designed to give cells better topographic cues during nerve regeneration.^15^ Among the materials employed, synthetic polymers are widely applied in the field of peripheral nerve regeneration because of their improved mechanical properties and versatility.^2^ Polyesters consisting of poly(lactic-*co*-glycolic acid) (PLGA) and poly(lactic acid) (PLA) have been commonly used to design various types of scaffolds for nervous tissue regeneration, especially for their attractive properties of high biocompatibility, excellent biodegradability, and ease of fabrication.^16−22^

The aim of the present work was to develop flexible and mechanically resistant platforms as a support and guide for neural cells during the regeneration process upon PNI. In the present work, a multichannel PLGA/poly(d,l-lactic acid) (PDLLA)/poly(ethylene glycol) 400 (PEG)-based scaffold was obtained through a sacrificial molding technique. The concentration of each scaffold component was chosen by means of a design of experiment approach (DoE). The developed multichannel scaffolds were finally characterized for mechanical and biopharmaceutical properties.

## 2 Experimental Section

### 2.1 Materials

Resomer RG 503H (poly(d,l-lactide-*co*-glycolide) 50:50, PM 24000-38000, PLGA), poly(ethylene glycol) (PEG) 400 Da, alginic acid sodium salt (ALG, medium viscosity grade), poly(ethylene oxide) (PEO) 600 kDa, Kolliphor P407 (POLOX), and ethyl acetate were purchased from Merck Life Science S.r.l. (Milan, Italy). Resomer R207 S (poly(d,l-lactic acid), PDLLA) was purchased from Darmstadt (Germany) and poly(ethylene oxide) (PEO) 4000 kDa from Colorcon (U.K.). Merck Life Science S.r.l. (Milan, Italy) was the supplier of all of the materials used for studies on the cell line.

### 2.2 Methods

#### 2.2.1 Preparation of PLGA-Based Films

As for the preparation of PLGA/PDLLA/PEG-based films, a concentration of PLGA (1.5% w/v) was selected and maintained constant, while different concentrations of PDLLA and PEG were employed to obtain 10 different formulations, as indicated in Table 1. In detail, the polymers were solubilized in ethyl acetate, and subsequently, 7 mL of the solution was poured into a glass Petri dish with a diameter of 5 cm. The solvent was allowed to evaporate under a flow hood at room temperature for 24 h. PLGA concentration was chosen on the basis of the results of our previous experiments. They were focused on the evaluation of the mechanical properties of PLGA films containing increasing concentrations of PLGA. A PLGA concentration of 1.5% w/v allowed us to obtain the best film in terms of detachability from the mold, mechanical resistance, and easy handling (data not shown). PDLLA was added to enhance film mechanical strength; for this reason, lower concentrations than that of PLGA were investigated, as PLGA should be the main component of the films. The concentrations of PEG were chosen on the basis of literature data.^23^

**Table 1:** Quali-Quantitative Composition of the Films

	concentrations		
name	PLGA (%w/v)	PDLLA (%w/v)	PEG (%w/w)^a^
film 1	1.5		
film 2	1.5	0.5	
film 3	1.5		20
film 4	1.5	0.5	20
film 5	1.5	0.75	20
film 6	1.5		10
film 7	1.5	0.5	10
film 8	1.5	0.75	10
film 9	1.5	0.375	10
film 10	1.5	0.375	20

a PEG concentration is expressed as a percent in weight (% w/w) of the total amount of the mixture PLGA and PDLLA.

##### 2.2.1.1 Characterization of the Mechanical Properties of PLGA-Based Films

The mechanical properties of films were investigated by means of a TA.XT plus Texture Analyzer equipped with a 5 kg load cell. Briefly, a 1 × 3 cm^2^ sample was clamped on an A/TG tensile grips probe, setting an initial distance of 1 cm between the grips. The upper grip was lifted at a constant speed of 0.5 mm/s. A final distance of 100 mm was fixed. Film thickness was measured by means of a Sicutool 3955G-50 (Milan, Italy) apparatus. The following parameters were calculated: maximum tensile strength (MPa) and elongation at break %. In the case of maximum tensile strength, the data obtained were normalized for the cross-sectional area, calculated by multiplying the film thickness (about 0.10 mm) by its width; six replicates were considered for each sample.^24^

##### 2.2.1.2 Design of Experiments

An experimental design was performed to evaluate the contribution of different parameters on the quality of the films, to obtain a coating layer that can provide the best mechanical properties to the scaffolds. Mean maximum tensile strength (TS) and elongation percent at break (EB%) were selected as response variables. For each experiment, two different batches of each formulation and six replicates were studied. The factors evaluated were PDLLA and PEG concentrations. A central composite design was chosen, as shown in Table 2. Chemometric Agile Tool software was used for data processing.

**Table 2:** Experimental Plan

name	PDLLA conc. (%w/v)	PEG conc. (%w/w)	PDLLA	PEG
film 1			–1	–1
film 2	0.5		0.33	–1
film 3		20	–1	1
film 4	0.5	20	0.33	1
film 5	0.75	20	1	1
film 6		10	–1	0
film 7	0.5	10	0.33	0
film 8	0.75	10	1	0
film 9	0.375	10	0	0
film 10	0.375	20	0	1

#### 2.2.2 Preparation of the Polymeric Solution to be Electrospun

The polymer solution composed of ALG medium viscosity grade, PEO, and POLOX was prepared in MilliQ water according to the following composition (% w/w): 1% w/w ALG, 1% w/w PEO 600 kDa, 2.2% w/w PEO 4000 kDa, and 2% w/w POLOX. PEO at two different grades, high- and low-molecular-weight, was used to enhance and facilitate the solution electrospinnability, and poloxamer was added to reduce the solution surface tension.^25,26^

#### 2.2.3 Electrospinning Process to Obtain Random and Aligned Microfibers

Freely water-soluble fibers (Fbs), random (FbsR) and aligned (FbsA), were obtained by electrospinning the polymeric solution based on ALG, PEO, and POLOX, using the apparatus STKIT-40 Linari Engineering (Grosseto, Italy) equipped with a flat collector and a rotary drum (⌀: 8 cm). In particular, the solution was pumped through a 21-gauge needle with a length of 15 mm. As for random fibers, the flat collector was used, and the process parameters were as follows: 25 cm (spinneret–collector distance), 20 kV (applied voltage), and 0.793 mL/h (flow rate). As for aligned fibers, the rotary drum was employed, and the process parameters were fixed at 15 cm, 20 kV, and 0.3965 mL/h; the cylindrical collector rotation frequency was kept at 200 Hz. Environmental parameters, temperature and relative humidity, were maintained in a range of 30–35 °C and 15–20%, respectively, for both processes. The solution was electrospun for 40 min to obtain FbR, while it was electrospun for 1.30 h to achieve FbA.

##### 2.2.3.1 Characterization of Fbs Morphological Properties and Dimensional Analysis

Fibers (FbsR, FbsA) were subjected to scanning electron microscopy analysis (Tescan Mira3 XMU, Brno, Czech Republic). The fiber mean diameter was measured using the imaging analysis program ImageJ 2.0.^27^

#### 2.2.4 Preparation of the Multichannel Scaffold

Regarding the preparation of the multichannel scaffold, as the first step Fbs were coated with a polymer blend. Particularly, FbsR and FbsA were soaked in a Petri dish containing an ethyl acetate solution composed of 1.5% w/v PLGA 50:50, 0.375% w/v PDLLA, and 0.125% w/w PEG. Then, the solvent was allowed to evaporate at room temperature under a flow hood, and as a result, a fiber-containing matrix was obtained (c-FbsR and c-FbsA), derived from the deposition of a thin polymer (PLGA/PDLLA/PEG) layer onto fibers. Afterward, the resulting coated fibers were detached from the Petri dish and soaked in MilliQ water for 1.30 h. This final step allowed us to obtain the complete dissolution of the fibers to achieve a platform characterized by the presence of inner random or aligned empty channels (MCR; MCA), surrounded and sustained by the polymer framework constituted by the polymer blend. During this step, PEG present in the coating partially dissolves, forming pores in the coating layer. This porosity should allow the entrance of water into the scaffold and contributes to fiber dissolution.

#### 2.2.5 Characterization of Multichannel Scaffolds

##### 2.2.5.1 Morphological and Dimensional Analysis Evaluation

Multichannel scaffolds (MCR and MCA) were subjected to scanning electron microscopy analysis (Tescan Mira3 XMU, Brno, Czech Republic); channel size was measured using the imaging analysis program ImageJ 2.0.

##### 2.2.5.2 Mechanical Properties Evaluation

The mechanical properties of coated fibers (c-FbsR and c-FbsA) and multichannel scaffolds (MCR, MCA) were assessed by means of a TA.XT plus Texture Analyzer, equipped with a 5 kg load cell, as described in Paragraph 2.2.1.1. A final distance of 20 mm was fixed. MC thickness was measured by means of a Sicutool 3955G-50 (Milan, Italy) apparatus. The TS (MPa) and EB% were calculated for each MC. In the case of TS, the data obtained were normalized for the cross-sectional area, calculated by multiplying the c-Fbs and MC thickness (about 0.4 mm) for their width; six replicates were considered for each sample.

##### 2.2.5.3 Suture Retention Strength Evaluation

MCR and MCA suture retention strengths were measured by means of a TA.XT plus Texture Analyzer equipped with a 5 kg load cell. The suture retention strength was defined as the peak force reached during suture pull-out. It is related to the difficulty of the suturing operation and the stability of the suture connection.^28^ Square samples of 1.5 × 1.5 cm^2^ were obtained and used for the analysis. In detail, one end of the sample was clamped on the lower grip probe at 4 mm of sample length; a loop of a 2/0 nylon monofilament suture (Scicalife) was placed in the middle of the sample, namely, at 7.5 mm from each side of the sample and clamped on the upper grip probe (placed at 5 cm distance from the lower one). The suture retention strength was defined as the peak force obtained during the procedure. Six replicates were analyzed for both random and aligned MC.

##### 2.2.5.4 Degradation Test

Specimens of 1 × 3 cm^2^ were prepared as described in Section 2.2.4 and were maintained in PBS for 28 days in a heating/shaking water bath (FALC Instruments, Treviglio, Italy) (100 rpm) at 37 °C. At different time points (0, 7, 28 days), sample mechanical properties in terms of TS (MPa) and EB% were evaluated using a TA.XT plus Texture Analyzer, as described in Subsection 2.2.5.2. Moreover, a morphological evaluation was carried out by means of scanning electron microscopy (SEM) (Tescan Mira3 XMU, Brno, Czech Republic), and channel size was measured using the imaging analysis program ImageJ 2.0.

##### 2.2.5.5 In Vitro Indirect Biocompatibility and Adhesion Properties

The biocompatibility and adhesion properties of multichannel scaffolds were assessed on rat Schwann RT4-D6P2T cells (CRL-2768) (SCs) obtained from American Type Culture Collection (ATCC). The cells were cultured in polystyrene flasks in ATCC-formulated Dulbecco’s Modified Eagle’s Medium (DMEM), added with 10% v/v heat-inactivated Fetal Bovine Serum (FBS) and 1% v/v antibiotic–antimycotic solution. As for biocompatibility, the cells were incubated at 37 °C in a 5% CO_2_ atmosphere. The cells (p2–p8) were seeded in a 96-well plate (17,500 cells/cm^2^); after 24 h, the samples were added to each well and 24 h contact with the cells was maintained.^27^ In detail, as for the cytotoxicity test, the preparation of MCR and MCA was performed under sterile conditions: they were prepared under a laminar flow hood and then sterilized through ultraviolet (UV) irradiation for 24 h. Subsequently, the samples were placed in cryo vials and left in contact with complete medium (DMEM + 10% FBS + 1% antibiotic–antimycotic). After 7 days of contact, the conditioned medium was collected from each sample and placed in a 96-well plate previously seeded with SCs for 24 h. Complete medium (CM) was utilized as a reference. Finally, the MTT assay was performed. Briefly, after the removal of samples, each well was rinsed with phosphate-buffered saline (PBS), and 150 μL of 1 mg/mL MTT in DMEM without phenol red was added and incubated for 3 h (37 °C and 5% CO_2_). Finally, 100 μL of dimethyl sulfoxide (DMSO) was put in each well to obtain the complete dissolution of formazan crystals derived from MTT dye reduction by mitochondrial dehydrogenases of living cells. The solution absorbance was measured at 570 and 690 nm wavelengths after 60 s of mild shaking (100 rpm) (FLUOstar Omega Microplate Reader, BMG LabTech, Ortenberg, Germany). The results were expressed as % cell viability by normalizing the absorbance measured after contact with each sample with that measured for CM, used as reference. Six replicates were performed for each sample.^29^

As for cell adhesion properties, sample preparation and cell culturing methods were the same as the biocompatibility assay. The samples were placed in a 96-well plate and subsequently seeded with 50 μL of Schwann cells (17,5000 cells/well) to promote initial cell adhesion. Thereafter, the cells were maintained in an incubator at 37 °C in a 5% CO_2_ atmosphere with 95% relative humidity for 1 h. After that, another 150 μL of CM was added to each well (controls included), and the 96-well plates were left in an incubator for 3 and 7 days. Six replicates were performed for each sample; CM was used as a reference.

Cell distribution into MCR and MCA (at 3 and 7 days of culture) was appreciated by means of confocal laser scanning microscopy (CLSM, Leica TCS SP2, Leica Microsystems, Milan, Italy). After removing the medium, each well was rinsed with PBS, and then the cells adhered on MCs were fixed with 3% v/v glutaraldehyde solution in PBS for 1 h. Afterward, cell walls were permeabilized by means of a solution of 100 μL of Triton X-100 in PBS for 5 min, and then cellular cytoskeletons were stained by incubating with 50 μL of fluorescein isothiocyanate (FITC Atto 488) at 20 μg/mL in PBS for 45 min at room temperature.^30,31^ Then, each well was washed twice with PBS, and cell nuclei were stained with 50 μL of bisbenzimide H334342 trihydrochloride (HOECHST) diluted 1:10,000 in PBS for 10 min. Finally, the samples were mounted on a microscope slide covered using coverslips and analyzed with λ_ex_ = 350 nm and λ_em_ = 470 nm for HOECHST and λ_ex_ = 495 nm and λ_em_ = 519 nm for FITC. The acquired images were processed with the software associated with the microscope (Leica Microsystem, Milan, Italy).

##### 2.2.5.6 In Vivo Biocompatibility Evaluation

All animal experiments were performed in full accordance with the standard international ethical guidelines (European Communities Council Directive 2010/63/EU) approved by Italian Health Ministry (D.L. 116/92). The protocol followed was approved by the Local Institutional Ethics Committee of the University of Pavia for the use of animals and by Istituto Superiore di Sanità (ISS).^32^ In detail, four male rats (Wistar 200–250 g, Envigo RMS S.r.l.) were subjected to anesthetic treatment with equitensine at 3 mL/kg (39 mM pentobarbital, 256 mM chloral hydrate, 86 mM MgSO_4_, 10% v/v ethanol, and 39.6% v/v propylene glycol), and their back was shaved to remove all hair. In view of the in vivo application, the MCA specimen was selected as the sample to be evaluated. The preparation of MCA was carried out under a laminar flow hood. MCA samples were then cut with a biopsy punch to have a diameter of 4 mm and sterilized through UV irradiation for 24 h, before their usage. Afterward, they were subcutaneously implanted in an 8 mm incision performed in each rat’s back. The incisions were then sutured using strips (Steri-Strip Suture, Italy). Full-thickness biopsies were collected in accordance with the incisions 17 days after implantation, and a histological analysis was carried out. Additionally, a biopsy of healthy skin was collected for comparison.

Hematoxylin and eosin (H&E) was used to stain some sections, while picrosirius red (PSR) was used on others. Following deparaffinization, the sections were hydrated, lightly stained with Weigert’s hematoxylin to identify the nuclei, and then stained with PSR (1 h). The PSR polarization method is one of the best techniques of collagen histochemistry, and it is particularly useful to point out the organization and heterogeneity of collagen fiber in different connective tissues. Polarizing light assessment of PSR stain identified the old thick collagen I fibers as orange-to-red and the newly deposed, rich in collagen III fibers, as green.

Following that, after dehydration, xylene was used to clean each section, which was then mounted with DPX mounting medium. Stained sections were observed with a light microscope (Carl Zeiss Axiophot). A microscope digital 5 megapixels CCD camera Nikon DS - Fi2 was used to capture the images.

#### 2.2.6 Statistical Analysis

Experimental data obtained from the various measures were subjected to statistical analysis, performed by means of an Astatsa statistical calculator; one-way analysis of variance (ANOVA) was followed by Scheffe post hoc comparisons (*p* < 0.05).

## 3 Results and Discussion

### 3.1 Identification of Film Composition by means of a DoE Approach

The solvent casting technique was used to obtain polymer films, as it is a commonly used simple and cost-effective method.^33^ Ten films composed of PLGA, PDLLA, and PEG were prepared.

**Figure 1:**
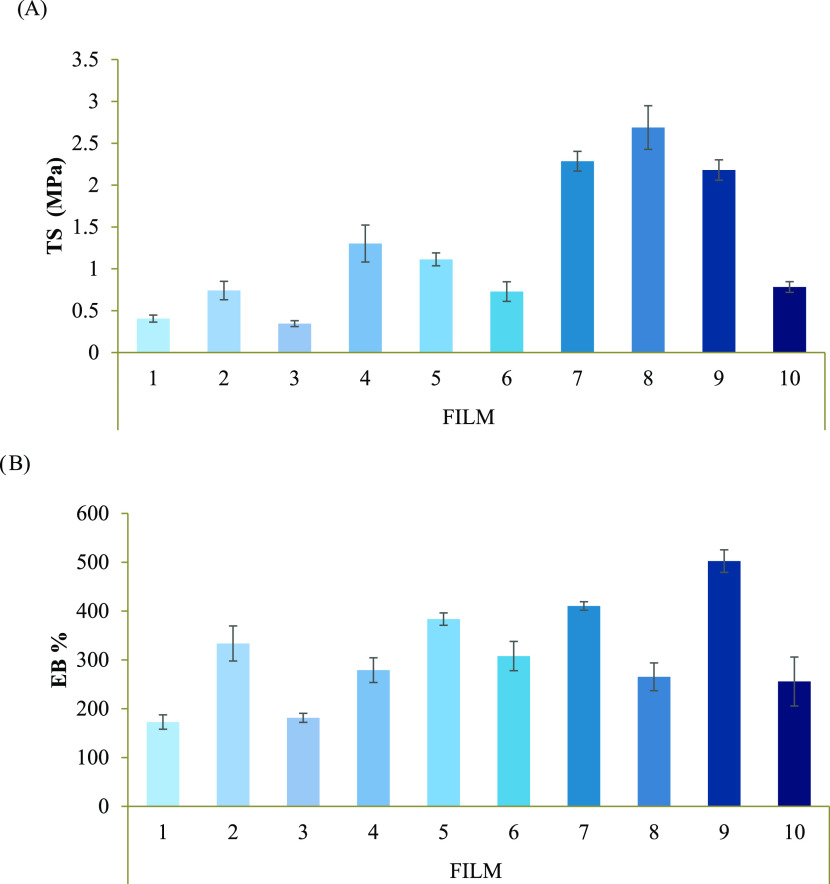
TS (A) and EB% (B) values resulting from the tensile test of films 1 to 10 characterized by the following polymer composition: film 1 (PLGA 1.5% w/v), film 2 (PLGA 1.5% w/v, PDLLA 0.5% w/v), film 3 (PLGA 1.5% w/v, PEG 20% w/w), film 4 (PLGA 1.5% w/v, PDLLA 0.5% w/v, PEG 20% w/w), film 5 (PLGA 1.5% w/v, PDLLA 0.75% w/v, PEG 20% w/w), film 6 (PLGA 1.5% w/v, PEG 10% w/w), film 7 (PLGA 1.5% w/v, PDLLA 0.5% w/v, PEG 10% w/w), film 8 (PLGA 1.5% w/v, PDLLA 0.75% w/v, PEG 10% w/w), film 9 (PLGA 1.5% w/v, PDLLA 0.375% w/v, PEG 10% w/w), and film 10 (PLGA 1.5% w/v, PDLLA 0.375% w/v, PEG 20% w/w) (mean values ± s.d.; *n* = 6).

Figure 1A,B shows the results obtained in terms of maximum resistance to traction or tensile strength (TS) and elongation percent at break (EB%), respectively, for all 10 films under investigation. It can be observed that films 7, 8, and 9, containing PDLLA (0.375, 0.5, and 0.75%) and PEG at 10%, are characterized by the highest values of TS. Moreover, films 7 and 9 show the highest values of EB%.

The DoE plan allowed us to investigate the influence of PDLLA and PEG levels on the mechanical properties of the films obtained. The addition of PDLLA to PLGA was considered, as PDLLA is recognized for its good mechanical properties, biodegradability, and biocompatibility.^34,35^ Moreover, PEG was added as a plasticizer to enhance film plasticity.^36^ Since a Central Composite Design with two factors required 10 experiments, that is, a number of experiments suitable for the purposes of the present study, it was decided to apply the Surface Response Methodology for quantitative modeling of the responses of our concern, as reported in Table 2. TS and EB% were chosen as the experimental response variables. The canonical quadratic model, *y* = *b*_0_ + *b*_1_·PDLLA + *b*_2_·PEG + *b*_12_·PDLLA·PEG + *b*_11_·PDLLA^2^ + *b*_12_·PEG^2^, was found as the best-fitting model explaining the results obtained.

The model equations computed for the two responses are
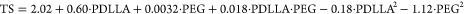

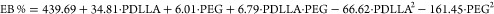
The coefficients of determination (*R*^2^) for these models were 0.889 and 0.907, respectively. The fitting quality of the models was considered sufficient for the purpose of the present study, considering the type of experiments involved.

**Figure 2:**
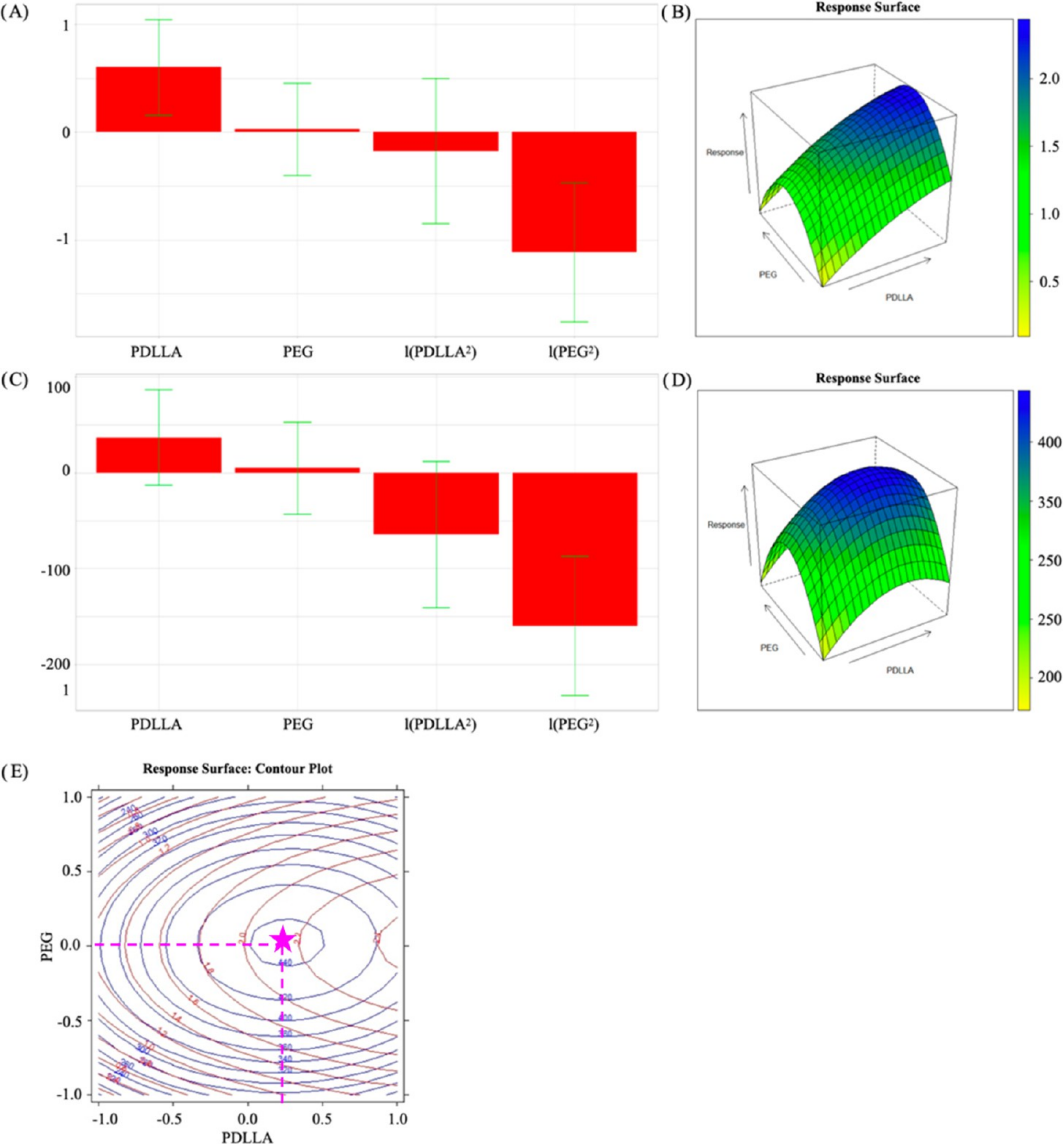
Results of the DoE model obtained for the 10 films analyzed. Coefficient bar chart (A) and the 3D contour plot (B) of the model for the response variable TS. Coefficient bar chart (C) and the 3D contour plot (D) of the model for the response variable EB%; superimposition of the 2D contour plots of the two response variables considered for the construction of the model, namely, TS (red) and EB% (blue) (E).

Figure 2A–D shows the coefficient bar charts together with the three-dimensional (3D) contour plots of the model responses for the variables considered. In Figure 2A,C, the coefficient values and their signs are reported together with the confidence intervals (at 95% probability level).

It can be noticed that PDLLA concentration has a significant positive effect only on TS, meaning that TS increases on increasing PDLLA concentration. As for PEG, the model points out the significant (quadratic) negative dependence of both TS and EB% on PEG, evidencing a parabolic behavior (Figure 2B,D).

In Figure 2E, the two-dimensional (2D) contour plots of the responses overlapped are shown. A region of the experimental domain, characterized by maximum values of both TS and EB%, was identified. Within the region where both responses reach the highest values, the formulation PDLLA 0.25, PEG 0, which corresponds to the composition approximately equal to PDLLA 0.375% w/v and PEG 12.5% w/w, was chosen as the optimal one.

Such formulation was characterized as those employed for the DoE, and the following results were obtained: EB% value of 498 ± 21 (mean values ± s.d.), namely, the highest value among all of the films considered. The value of EB% experimentally obtained by means of a Texture Analyzer was compared with that predicted by the model, and no significant difference was observed (444 ± 77, predicted mean value ± confidence interval at the 95% of probability).

Moreover, the TS of the same film was also measured; it was found to be 1.8 ± 0.3 MPa (mean value ± s.d.). This value was not significantly different from that predicted by the model (2.2 ± 0.7; mean value ± confidence interval at the 95% of probability). Therefore, the models for TS and EB% proved to be validated, as the differences between experimental and theoretically predicted values are not statistically significant. As conclusive experimental evidence, the film derived from the optimization study showed the best equilibrium between the two properties considered, and for this reason, it was chosen as the best candidate for the prosecution of the work.

### 3.2 Morphological Characterization of ALG-Based Fibers

The solution based on ALG and PEO was subjected to an electrospinning process, setting the process parameters so as to obtain fibers (Fbs) with a mean diameter in the microsize range. A diameter in the range of micrometers is particularly desirable for the administration here considered, mainly because it is reported in the literature that microchannels can provide a more accurate guide for neuronal cells during the regeneration process.^10,37^ It is recognized that cell behaviors, including morphology, migration, proliferation, and differentiation, are affected by the electrospun topographical and morphological fiber properties;^38,39^ the extracellular matrix (ECM) in many tissues possesses an oriented structure, responsible for peculiar mechanical properties related to specific functions. The orientation of fibers or channels into scaffolds is intended to mimic the ECM architecture.^38^ Because of the importance of fiber/channels’ mean diameter and orientation, herein both random fibers with a major diameter and aligned fibers with an inferior diameter were developed to obtain random or aligned microchannels and thus investigate the effect of these two parameters on scaffold properties.

**Figure 3:**
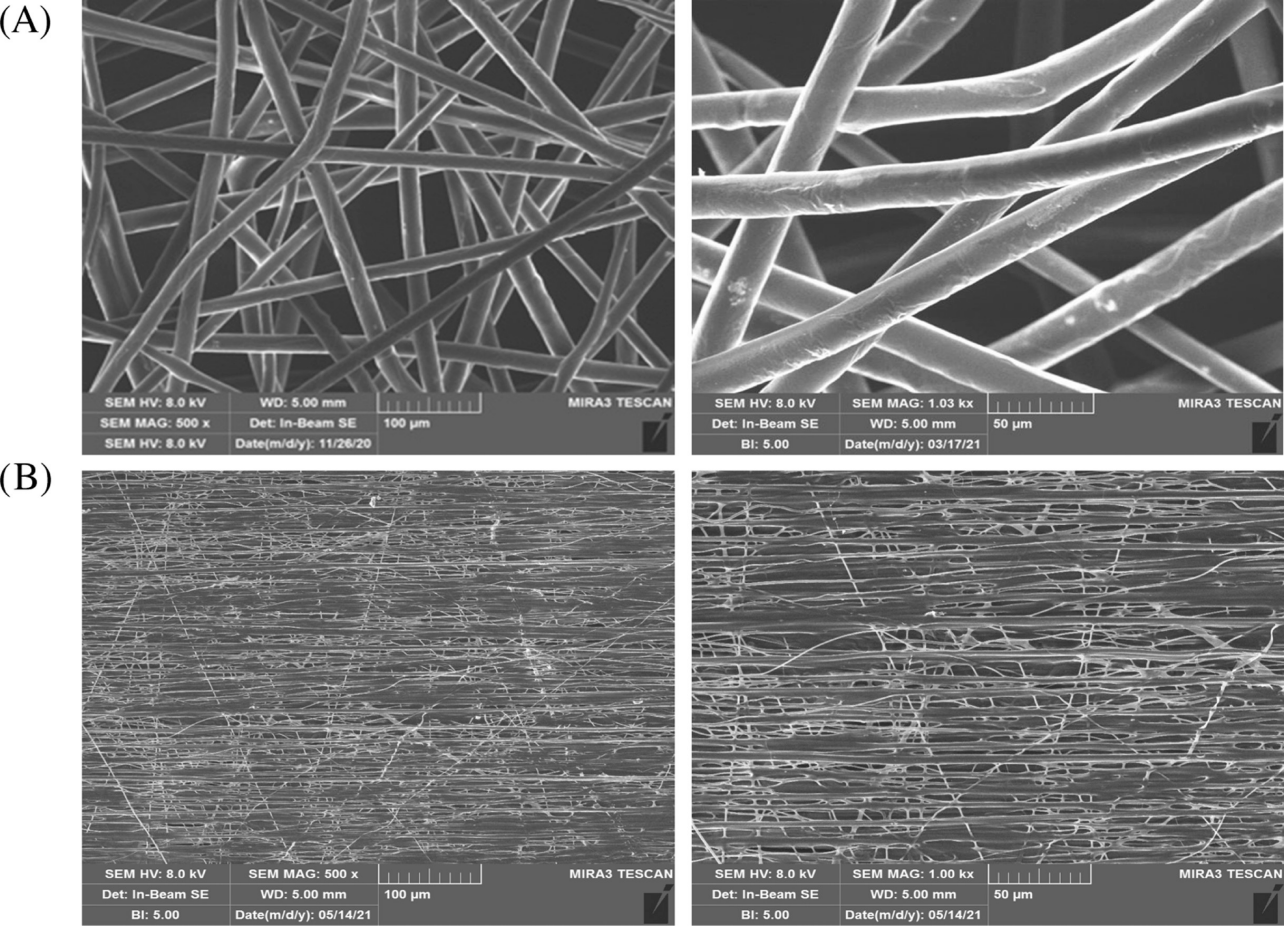
SEM micrographs of FbsR (A) and FbsA (B) at two different magnifications: magnification at 1k× for a more detailed view of the morphology of both fibers, and magnification at 500× for a more general view of the 3D network of both fibers.

Electrospun Fbs, random (FbsR) and aligned (FbsA), were obtained and characterized for morphological properties. Figure 3 shows an SEM microphotograph of FbsR and FbsA. FbsR are characterized by a mean diameter of 22.15 ± 2.45 μm, whereas the mean diameter of FbsA is 5.55 ± 2.45 μm (mean values ± s.d.). Both the fibrous scaffolds obtained, as can be observed in the micrographs, are homogeneous, free of beads, and characterized by a smooth and regular surface. The mean diameter obtained, namely, in the range of 5–20 μm, is suitable to obtain microchannels that should provide a guide for nervous cells, as reported in the literature.^9^

### 3.3 Characterization of Multichannel Scaffolds

#### 3.3.1 Morphological Properties

**Figure 4:**
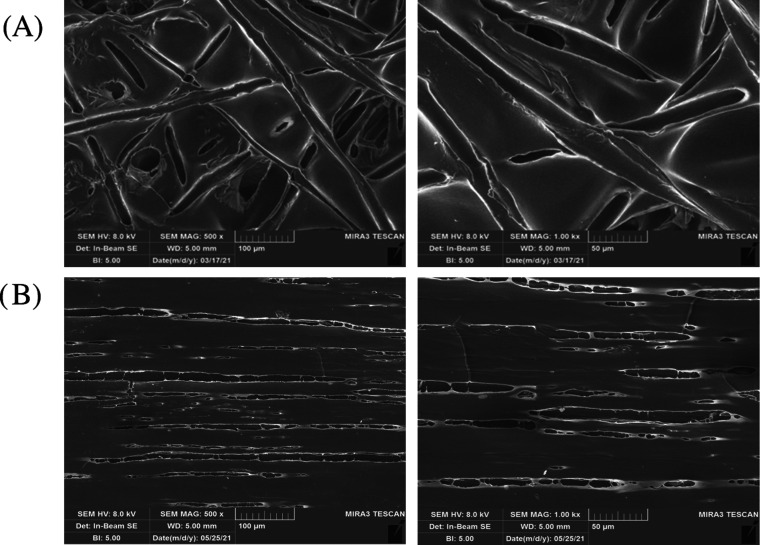
SEM micrographs at two different magnifications (500x and 1kx) of MCR (A) and MCA (B) clearly showing the presence of empty channels within the structure of the polymeric platform and the different orientation of such channels derived from the different disposition of electrospun fibers (random or aligned) from which channels originated.

Multichannel NGCs have been widely investigated and studied, especially for the easiness of production and for their tunable properties.^39−41^ The multichannel scaffold (MC) developed in the present work was obtained by a multistep process starting from Fbs coating, followed by the dissolution of the inner component consisting of hydrophilic fibers by immersion in water. Therefore, scaffolds with random or aligned empty channels (MCR; MCA), based on the PLGA/PDLLA/PEG framework, was achieved. Figure 4 shows SEM images at two different magnifications (500× and 1k×) of MCR and MCA obtained; their mean diameters were 16 ± 3 for MCR and 8 ± 2 (mean values ± s.d.) for MCA. The mean diameter of empty channels generated after fiber dissolution was significantly different (ANOVA one way; post hoc Scheffè test (*p* value ≤ 0.05)) from the initial mean diameter of fibers, probably due to a partial relaxation of the structure following solubilization of the inner fibrillar structure. Despite that, the interconnected network typical of fibers can be easily recognized from SEM images reported; it is possible to appreciate the morphology of the multichannel scaffold, which appears as random (A) or aligned (B) voids the channels generated after fiber dissolution surrounded and sustained by the PLGA/PDLLA/PEG polymeric matrix. Moreover, on a comparable area, MCA appears to be characterized by a lower porosity with respect to MCR.

#### 3.3.2 Mechanical Properties

**Figure 5:**
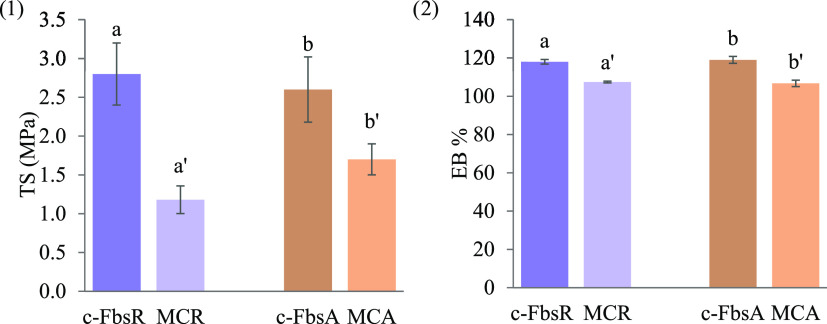
Mechanical properties of coated fibers (c-FbsR and c-FbsA) and multichannel scaffolds (MCR and MCA) subjected to a tensile test: TS (MPa) (1) and EB% (2) values (mean values ± s.d.; *n* = 10). ANOVA one way; post hoc Scheffè test (*p* ≤ 0.05); regarding 5 (1): a vs a′, b′; a′ vs b, b′; b vs b′; regarding 5 (2): a vs a′, b′; a′ vs b; b vs b′.

Both coated fibers (c-FbsR and c-FbsA) and multichannel scaffolds (MCR and MCA) were subjected to a tensile stress test to evaluate and compare their mechanical properties. Figure 5 shows the comparison between the mechanical properties of the samples in terms of TS (MPa) (1) and EB% (2). Since scaffolds could be characterized by different thicknesses, TS values reported were divided for the cross-sectional area of the scaffold exposed to the test to normalize the results obtained. First, it has to be highlighted that the significantly lower values of TS and EB% observed for MCs with respect to the relevant coated fibers demonstrate the presence of empty channels within the scaffolds. Moreover, regarding TS, a mean value for MCA higher than that for MCR was observed. This is probably due to the fact that MCA is characterized by channels with smaller sizes and by a lower porosity with respect to MCR.

Indeed, no statistical differences were observed between EB% values of MCR and MCA, indicating that the elastic behavior of the scaffolds does not depend on the inner channel orientation. The results obtained indicate that both scaffolds are characterized by good mechanical properties in terms of mechanical resistance and plasticity. In particular, both scaffolds are able to elongate without breaking for a distance corresponding to more than 100% of their initial length, indicating an optimal capacity to adapt and withstand tension and compression when inserted in the nerve injury site.

Synthetic biodegradable polymers used to obtain the external matrix proved to grant good mechanical properties to the scaffold developed. TS values are in line with the data reported in the relevant literature.^10,40^

#### 3.3.3 Suture Retention Strength

The behavior of the multichannel scaffolds when subjected to a suturing process was also investigated. The thicknesses for all samples were very close to each other, with a value of about 0.4 mm. MCR is characterized by a suture retention strength of about 1.8 ± 0.5 N, which is comparable to that of epineurium, reported in the literature (∼2 N).^42^ The value of suture retention strength of MCA was significantly lower, namely, 0.91 ± 0.1 N. That can be explained by the fact that macroscopically, the MCA appears as a set of bundles joined together by the external polymeric matrix; such an ensemble of channels characterizing the internal structure of the scaffolds is aligned toward the direction of the traction during the test.

When the suture filament needle is anchored to MCA, slight separation of the bundle occurs, forming a little leak within the structure, and this could be the reason why the value of suture retention strength of MCA results is lower than that of MCR.

#### 3.3.4 Degradation Study

When using synthetic polymers, it is critical to evaluate the degradation rate of the scaffold developed to predict the preservation of the scaffold when applied in vivo. The scaffold has to maintain its morphological and mechanical properties with time, to grant a correct regeneration of the injury, but, on the other hand, it also has to be characterized by a proper degradation time, so as to be totally deteriorated once the injury has healed.^43^ Herein, in particular, PLGA and PDLLA were employed precisely because they are recognized for their optimal biodegradation properties.^16^

**Figure 6:**
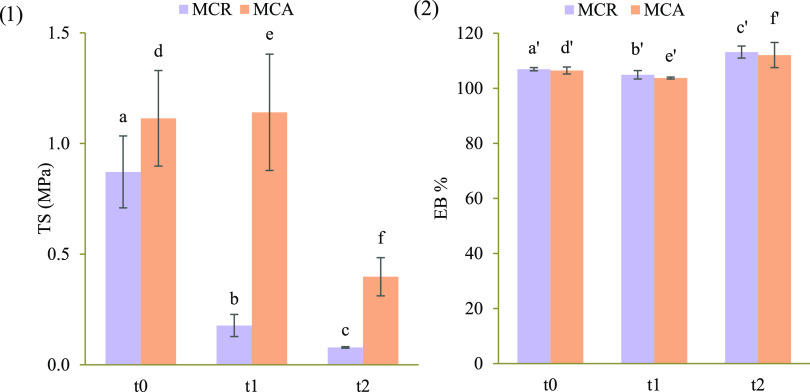
TS (MPa) (1) and EB% (2) values of MCR and MCA at different time points evaluated during the degradation study carried out in PBS medium (mean values ± s.d.; *n* = 3). ANOVA one way; post hoc Scheffè test (*p* ≤ 0.05); a vs b-c; b vs c; d vs e-f; a′ vs c′; b′ vs c′; e′ vs f′.

To assess MC properties with time, in terms of mechanical resistance, elasticity, and morphology, a degradation test was performed on MCR and MCA for a period of 28 days at 37 °C in PBS medium. Figure 6(1, 2) shows the comparison between TS and EB% at different time points (*t*_0_ = 0 days; *t*_1_ = 7 days; *t*_2_ = 28 days) for MCR and MCA. As for MCR, a marked decrease of TS values between *t*_0_ and *t*_1_ is observed, while it is less appreciable between *t*_1_ and *t*_2_. The same scaffold shows a significant increase in EB% after 7 days, as the progression of the degradation process could cause lower resistance to elongation, probably due to polymer chain relaxation. In the case of MCA, no statistically significant differences were observed between TS values measured at *t*_0_ and *t*_1_, whereas a statistically significant decrease of TS was observed after 7 days; this behavior could indicate a delay in the beginning of the degradation process. The difference in TS value reduction between MCR and MCA is related to the different channel sizes and porosity of the two scaffolds. It must be underlined that MCA is characterized by smaller channels and by a minor porosity with respect to MCR.

No statistically significant differences were observed between the EB% values of the scaffolds after the different biodegradation times considered, meaning that the plasticity properties did not change.

The results obtained indicate that MCA is more resistant to the degradation process in comparison to MCR.

**Figure 7:**
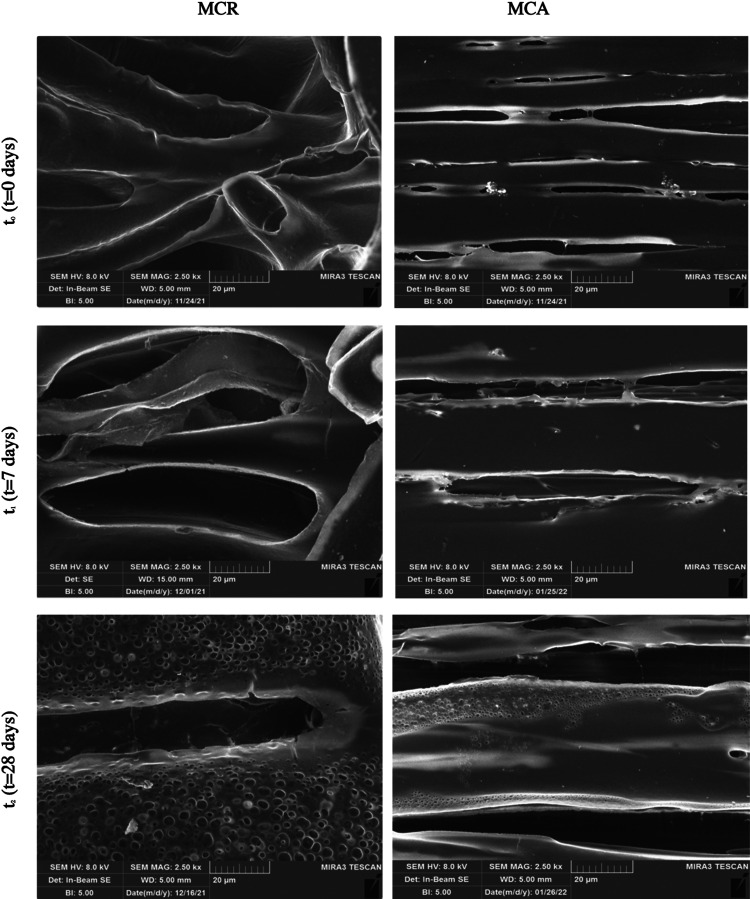
SEM images at 2.5k× magnification of MCR and MCA, respectively, at *t*_0_ (*t* = 0 days), *t*_1_ (*t* = 7 days), and *t*_2_ (*t* = 28 days).

**Figure 8:**
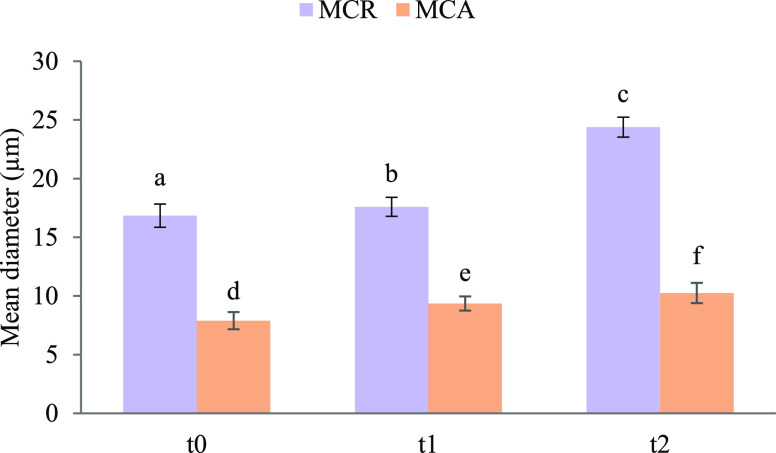
MCR and MCA mean diameters at each time point of the degradation study performed in PBS: *t*_0_ (0 days), *t*_1_ (7 days), and *t*_2_ (28 days). Mean diameter size evaluation with ImageJTM software (mean values ± s.d.; *n* = 30); ANOVA one way; post hoc Scheffè test (*p* ≤ 0.05): a vs c; b vs c.

Morphologic properties of the scaffolds were also evaluated at each time point considered for the degradation study (*t*_0_ = 0 days; *t*_1_ = 7 days; *t*_2_ = 28 days). Figure 7 shows SEM images of MCR and MCA at each time point, after drying under a flow hood. It is possible to appreciate the morphological changes related to the channels, more marked in the case of MCR, for which a faster coating degradation is observed when compared to the aligned one. Especially, at *t*_2_, small holes appear in the MCR polymeric matrix. This behavior is confirmed by the dimensional analysis of the channels during the degradation. Figure 8 shows channel diameter values (μm) of MCR and MCA at the different time points of the test. As can be noticed, MCR displays a progressive significant increase in the channel diameters, while this change is not significantly relevant in MCA.

#### 3.3.5 In Vitro Indirect Biocompatibility and Cell Adhesion

The cytotoxic effect and cell adhesion properties of MCR and MCA were investigated on Schwann cells, namely, the main neuroglia of the PNS and the most common cell line model for in vitro studies on scaffolds for PNI.^44^ Schwann cells are able to enhance nerve regeneration upon PNI, acting as matrix producers and growth factor providers. Moreover, after an injury that causes the axonal contact breakdown, axonal regrowth is promoted by Schwann cell differentiation and proliferation together with the downregulation of myelin-related genes and the upregulation of adhesion molecules, neurotrophins, cytokines, and their receptors.^45^

**Figure 9:**
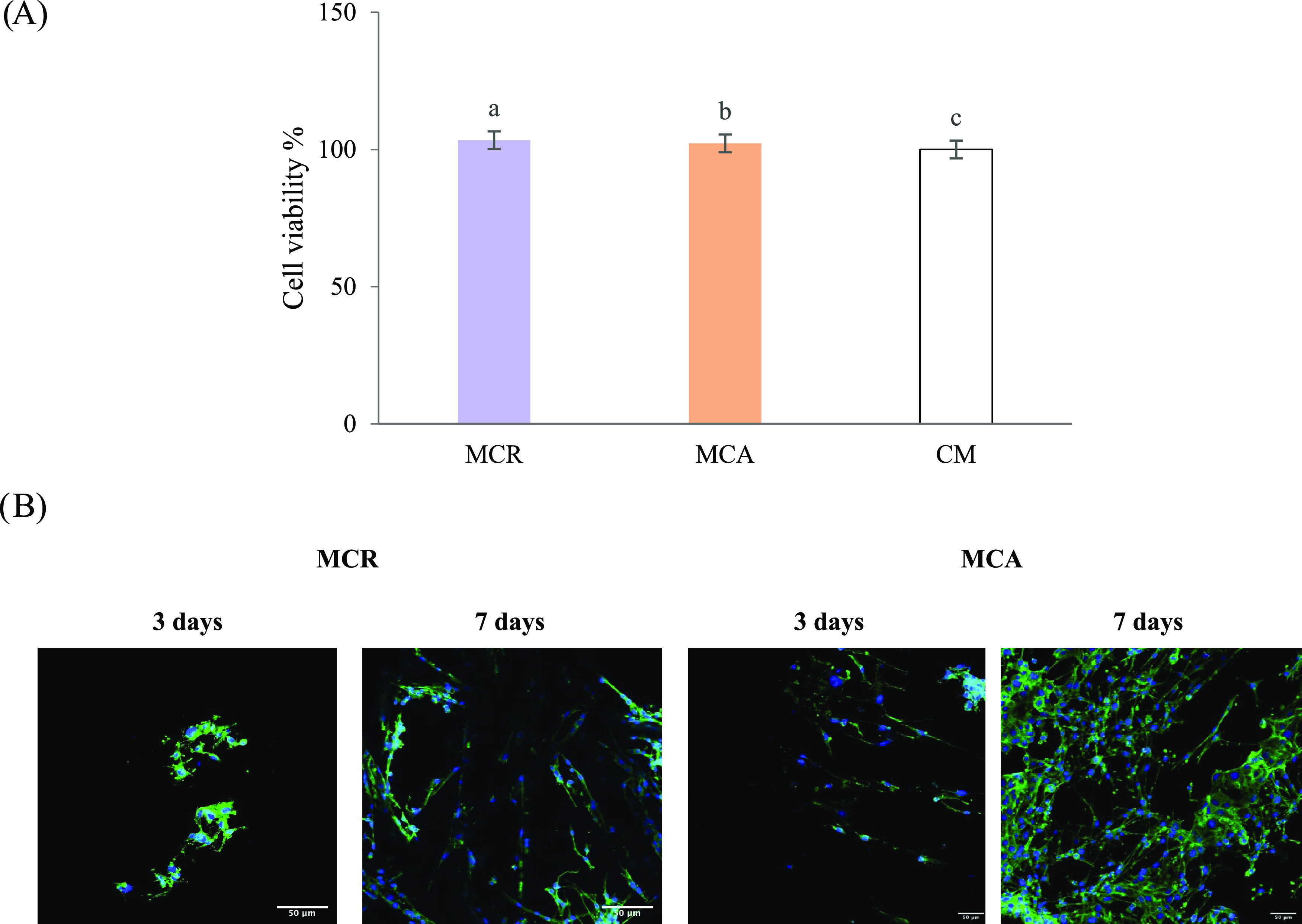
(A) Viability% values calculated after indirect contact of cells with MCR and MCA (mean values ± s.d.; *n* = 12). CM was considered as control. ANOVA one-way: no statistical difference. (B) CLSM microphotographs of Schwann cells grown on MCR and MCA scaffolds for 3 and 7 days: confocal laser scanning microscopy; nuclei are stained in blue, Hoechst 33258, while cytoskeleton is stained in green, FITC. Scale bars: 50 μm.

Results of the cytotoxicity test are reported in Figure 9A as the percentage of living cells after contact with the conditioned medium left in contact with the samples for 7 days. Both MCR and MCA can be considered highly biocompatible since they are characterized by cell viability % values not statistically different from that of the control (CM). Moreover, Figure 9B shows CLSM microphotographs of Schwann cells grown on a multichannel scaffold, after 3 and 7 days, during the cell adhesion assay. It is evident that cell proliferation on MCR was limited when compared to MCA. In fact, MCA microphotographs reveal that the cells proliferated and grew on and inside the polymeric matrix, resulting in complete colonization. It is clear from both the tests performed that the aligned channel pattern has a greater ability in supporting and facilitating cell adhesion and growth on the scaffolds, suggesting an optimal cell–substrate interaction. On the contrary, scaffolds random orientation channels are characterized by a poorer interaction with the cells, as indicated by the lower number of cells on the scaffold surface.

#### 3.3.6 In Vivo Scaffold Safety Study

**Figure 10:**
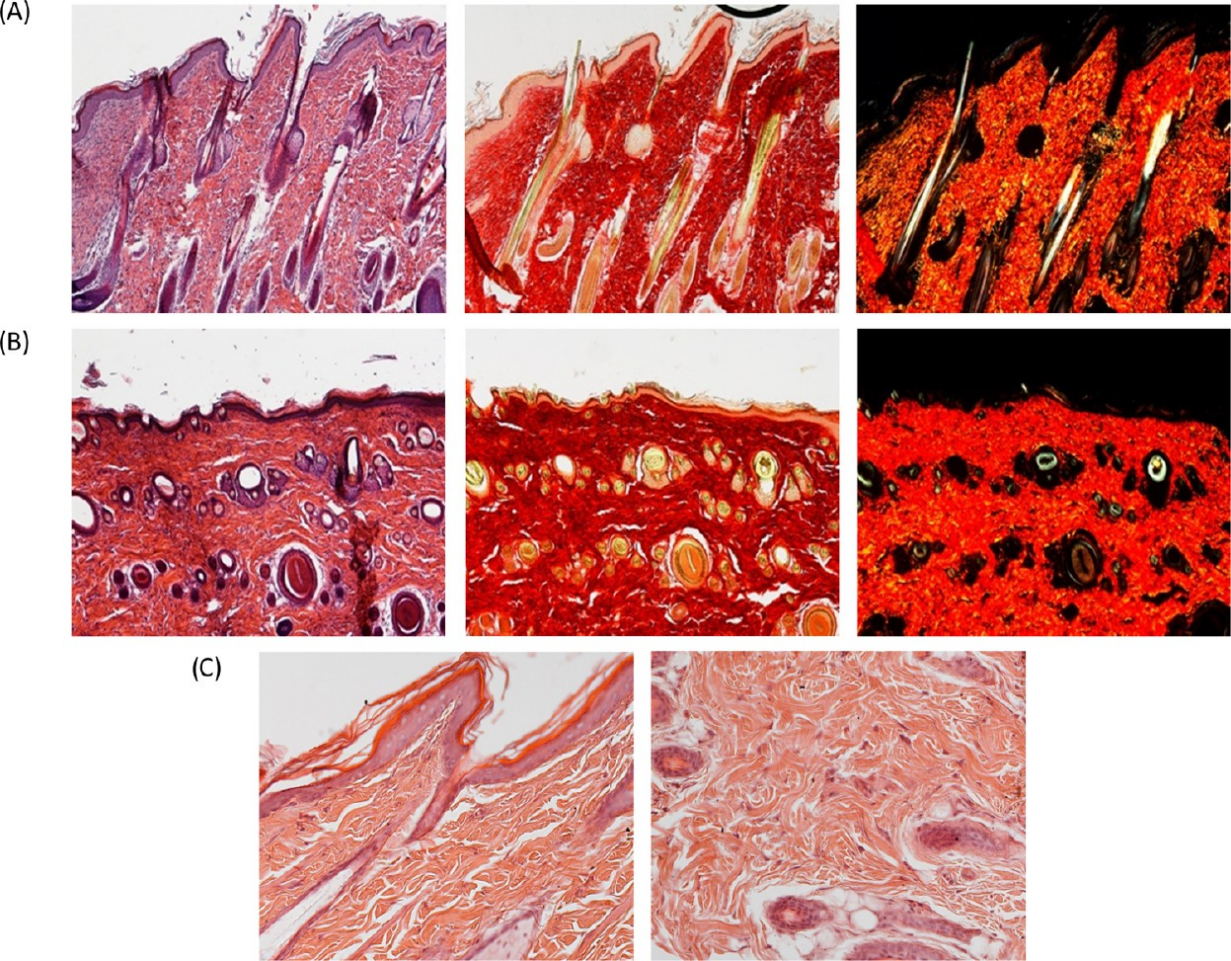
Hematoxylin and eosin (H&E) and PSR sections of (A) healthy skin (magnification 5×). (B) Subcutaneous implant of MCA (magnification 5×). (C) Left: healthy skin; right: subcutaneous implant of MCA (magnification 20×). Hematoxylin stains cellular nuclei in purplish blue, while eosin stains the extracellular matrix and the cytoplasm in pink. The stain’s overall color patterns provide an overview of the structure of the tissue sample and show the general layout and distribution of cells. Picrosirius stain binds specifically to collagen fibrils, revealing the organization and the heterogeneity of collagen fibrils within the tissue. Panels (A) and (B): each micrograph frame has a width of 1780 μm. Panel (C): each micrograph frame has a width of 430 μm.

Figure 10 shows a comparison of the H&E and PSR sections of intact skin and skin after subcutaneous implantation of MCA. The PRS polarization method is a histochemistry technique critically useful to reveal both organization and heterogeneity of collagen fibers in connective tissues. In detail, PSR stain is able to identify the old and newly deposed collagen type I fibers as orange-to-red filaments, while collagen type III fibers appear to be green.^46,47^

At 18 days following the MCA implant (Figure 10B), the wound area was difficult to recognize from the adjacent control skin. Complete regeneration of both epidermis and dermis was observed. In particular, a well-formed keratinized squamous epithelium was identified. PSR stain showed collagen fibers completely remodeled in the epidermis and dermis; hair follicles, and sebaceous glands were completely restored and outcoming hairs were present. No collagen type III fibers were observed, and only collagen type I orange-to-red fibers were present, with a pattern identical to that of intact skin, confirming the complete safety of the scaffold. As better seen at high magnification (C), 18 days after the MCA subcutaneous implant (left panel), the epidermis appears correctly reformed in the canonical layers (i.e., basal, spinous, granular, and corneous layers are clearly visible and well-defined). In the papillary and reticular dermis, no perivascular or other types of lymphocytes were visible. No cells as regards size or granular content suggesting the presence of macrophages was evidenced, and the vascular component, i.e., blood vessels, were also present in size and number comparable to those of the healthy skin (right panel).

## 4 Conclusions

In the present work, a multichannel scaffold based on a biodegradable polymer blend was successfully designed and developed. Fixing PLGA concentration, a DoE approach was exploited to investigate the influence of PDLLA and PEG concentration on the mechanical properties of the films obtained via solvent casting. The optimized models allowed us to find the best polymeric blend composition. Moreover, random and aligned electrospun microfibers, characterized by a mean diameter in the micrometer-range size, optimal for peripheral nerve application, were achieved and used as a sacrificial mold to obtain the final multichannel platforms through the multistep process developed here.

Through a simple and effective process, a promising innovative biodegradable, biocompatible, and safe microscale multichannel scaffold was obtained. The work is in progress to optimize the scaffold manufacturing process. A critical aspect that will be considered is the reproducibility of the inner structure in terms of the continuous channel pattern.

The overall results obtained indicate that the MCA platform is a promising candidate to provide a biomimetic environment to the site of injury, thanks to its aligned inner structure that can favor cell growth.

A future perspective is to employ the MCA as a hosting scaffold to load neuronal growth factors or cells, in view of further improving its biomimetic properties.

## References

[ref1] Ferrante M. A. The Assessment and Management of Peripheral Nerve Trauma. Curr. Treat. Options Neurol. 2018, 20, 25 10.1007/s11940-018-0507-4. 29855741

[ref2] Jiang H.; Qian Y.; Fan C.; Ouyang Y. Polymeric Guide Conduits for Peripheral Nerve Tissue Engineering. Front. Bioeng. Biotechnol. 2020, 8, 582646 10.3389/fbioe.2020.582646. 33102465 PMC7546820

[ref3] Sullivan R.; Dailey T.; Duncan K.; Abel N.; Borlongan C. Peripheral Nerve Injury: Stem Cell Therapy and Peripheral Nerve Transfer. Int. J. Mol. Sci. 2016, 17, 2101 10.3390/ijms17122101. 27983642 PMC5187901

[ref4] Han G. H.; Peng J.; Liu P.; Ding X.; Wei S.; Lu S.; Wang Y. Therapeutic Strategies for Peripheral Nerve Injury: Decellularized Nerve Conduits and Schwann Cell Transplantation. Neural Regener. Res. 2019, 14, 1343–1351. 10.4103/1673-5374.253511. PMC6524503 30964052

[ref5] Carvalho C. R.; Oliveira J. M.; Reis R. L. Modern Trends for Peripheral Nerve Repair and Regeneration: Beyond the Hollow Nerve Guidance Conduit. Front. Bioeng. Biotechnol. 2019, 7, 337 10.3389/fbioe.2019.00337. 31824934 PMC6882937

[ref6] Vigani B.; Rossi S.; Sandri G.; Bonferoni M. C.; Ferrari F. Design and Criteria of Electrospun Fibrous Scaffolds for the Treatment of Spinal Cord Injury. Neural Regener. Res. 2017, 12, 1786–1790. 10.4103/1673-5374.219029. PMC5745824 29239316

[ref7] Chen P.; Piao X.; Bonaldo P. Role of Macrophages in Wallerian Degeneration and Axonal Regeneration after Peripheral Nerve Injury. Acta Neuropathol. 2015, 130, 605–618. 10.1007/s00401-015-1482-4. 26419777

[ref8] Li R.; Li D.-h.; Zhang H.-y.; Wang J.; Li X.-k.; Xiao J. Growth Factors-Based Therapeutic Strategies and Their Underlying Signaling Mechanisms for Peripheral Nerve Regeneration. Acta Pharmacol. Sin. 2020, 41, 1289–1300. 10.1038/s41401-019-0338-1. 32123299 PMC7608263

[ref9] Wieringa P. A.; de Pinho A. R. G.; Micera S.; van Wezel R. J. A.; Moroni L. Biomimetic Architectures for Peripheral Nerve Repair: A Review of Biofabrication Strategies. Adv. Healthcare Mater. 2018, 7, 1701164 10.1002/adhm.201701164. 29349931

[ref10] Vijayavenkataraman S. Nerve Guide Conduits for Peripheral Nerve Injury Repair: A Review on Design, Materials and Fabrication Methods. Acta Biomater. 2020, 106, 54–69. 10.1016/j.actbio.2020.02.003. 32044456

[ref11] Ray W. Z.; Mackinnon S. E. Management of Nerve Gaps: Autografts, Allografts, Nerve Transfers, and End-to-Side Neurorrhaphy. Exp. Neurol. 2010, 223, 77–85. 10.1016/j.expneurol.2009.03.031. 19348799 PMC2849924

[ref12] Houshyar S.; Bhattacharyya A.; Shanks R. Peripheral Nerve Conduit: Materials and Structures. ACS Chem. Neurosci. 2019, 10, 3349–3365. 10.1021/acschemneuro.9b00203. 31273975

[ref13] Kolar M. K.; Kingham P. J. Peripheral Nerve Tissue Engineering. In Tissue Engineering Using Ceramics and Polymers, 3rd ed.; Elsevier Inc., 2022; pp 481–517.

[ref14] Kang N. U.; Lee S. J.; Gwak S. J. Fabrication Techniques of Nerve Guidance Conduits for Nerve Regeneration. Yonsei Med. J. 2022, 63, 114–123. 10.3349/ymj.2022.63.2.114. 35083896 PMC8819402

[ref15] Apablaza J. A.; Lezcano M. F.; Marquez A. L.; Sánchez K. G.; Oporto G. H.; Dias F. J. Main Morphological Characteristics of Tubular Polymeric Scaffolds to Promote Peripheral Nerve Regeneration—A Scoping Review. Polymers 2021, 13, 2563 10.3390/polym13152563. 34372166 PMC8347244

[ref16] Yang E. Z.; Zhang G. W.; Xu J. G.; Chen S.; Wang H.; Cao L. L.; Liang B.; Lian X. F. Multichannel Polymer Scaffold Seeded with Activated Schwann Cells and Bone Mesenchymal Stem Cells Improves Axonal Regeneration and Functional Recovery after Rat Spinal Cord Injury. Acta Pharmacol. Sin. 2017, 38, 623–637. 10.1038/aps.2017.11. 28392569 PMC5457698

[ref17] Xu F.; Zhang K.; Lv P.; Lu R.; Zheng L.; Zhao J. NECL1 Coated PLGA as Favorable Conduits for Repair of Injured Peripheral Nerve. Mater. Sci. Eng.: C 2017, 70, 1132–1140. 10.1016/j.msec.2016.03.043. 27772714

[ref18] Makadia H. K.; Siegel S. J. Poly Lactic-Co-Glycolic Acid (PLGA) as Biodegradable Controlled Drug Delivery Carrier. Polymers 2011, 3, 1377–1397. 10.3390/polym3031377. 22577513 PMC3347861

[ref19] Amini S.; Salehi H.; Setayeshmehr M.; Ghorbani M. Natural and Synthetic Polymeric Scaffolds Used in Peripheral Nerve Tissue Engineering: Advantages and Disadvantages. Polym. Adv. Technol. 2021, 32, 2267–2289. 10.1002/pat.5263.

[ref20] Amani H.; Kazerooni H.; Hassanpoor H.; Akbarzadeh A.; Pazoki-Toroudi H. Tailoring Synthetic Polymeric Biomaterials towards Nerve Tissue Engineering: A Review. Artif. Cells, Nanomed., Biotechnol. 2019, 47, 3524–3539. 10.1080/21691401.2019.1639723. 31437011

[ref21] Gregory H.; Phillips J. B. Materials for Peripheral Nerve Repair Constructs: Natural Proteins or Synthetic Polymers?. Neurochem. Int. 2021, 143, 104953 10.1016/j.neuint.2020.104953. 33388359

[ref22] Lasprilla A. J. R.; Martinez G. A. R.; Lunelli B. H.; Jardini A. L.; Filho R. M. Poly-Lactic Acid Synthesis for Application in Biomedical Devices - A Review. Biotechnol. Adv. 2012, 30, 321–328. 10.1016/j.biotechadv.2011.06.019. 21756992

[ref23] Bourtoom T. Plasticizer Effect on the Properties of Biodegradable Blend Film from Rice Starch-Chitosan. Songklanakarin J. Sci. Technol. 2008, 30, 149–155.

[ref24] Vigani B.; Rossi S.; Sandri G.; Bonferoni M. C.; Rui M.; Collina S.; Fagiani F.; Lanni C.; Ferrari F. Dual-Functioning Scaffolds for the Treatment of Spinal Cord Injury: Alginate Nanofibers Loaded with the Sigma 1 Receptor (S1R) Agonist RC-33 in Chitosan Films. Mar. Drugs 2020, 18, 21 10.3390/md18010021. PMC7024184 31887983

[ref25] Vigani B.; Valentino C.; Sandri G.; Listro R.; Fagiani F.; Collina S.; Lanni C.; Bonferoni M. C.; Caramella C. M.; Rossi S.; Ferrari F. A Composite Nanosystem as a Potential Tool for the Local Treatment of Glioblastoma: Chitosan-Coated Solid Lipid Nanoparticles Embedded in Electrospun Nanofibers. Polymers 2021, 13, 1371 10.3390/polym13091371. 33922214 PMC8122751

[ref26] Vigani B.; Rossi S.; Milanesi G.; Bonferoni M. C.; Sandri G.; Bruni G.; Ferrari F. Electrospun Alginate Fibers: Mixing of Two Different Poly(Ethylene Oxide) Grades to Improve Fiber Functional Properties. Nanomaterials 2018, 8, 971 10.3390/nano8120971. 30477265 PMC6315736

[ref27] Valentino C.; Vigani B.; Fedeli I.; Miele D.; Marrubini G.; Malavasi L.; Ferrari F.; Sandri G.; Rossi S. Development of Alginate-Spermidine Micro/Nanogels as Potential Antioxidant and Anti-Inflammatory Tool in Peripheral Nerve Injuries. Formulation Studies and Physico-Chemical Characterization. Int. J. Pharm. 2022, 626, 122168 10.1016/j.ijpharm.2022.122168. 36075525

[ref28] Yin A.; Bowlin G. L.; Luo R.; Zhang X.; Wang Y.; Mo X. Electrospun Silk Fibroin/Poly (L-Lactide-ε-Caplacton) Graft with Platelet-Rich Growth Factor for Inducing Smooth Muscle Cell Growth and Infiltration. Regener. Biomater. 2016, 3, 239–245. 10.1093/RB/RBW026. PMC4966297 27482466

[ref29] Vigani B.; Valentino C.; Sandri G.; Caramella C. M.; Ferrari F.; Rossi S. Spermidine Crosslinked Gellan Gum-Based “Hydrogel Nanofibers” as Potential Tool for the Treatment of Nervous Tissue Injuries: A Formulation Study. Int. J. Nanomed. 2022, 17, 3421–3439. 10.2147/IJN.S368960. PMC9356740 35942070

[ref30] Zhang L.; Yang X.; Yue Y.; Ye J.; Yao Y.; Fu Y.; Li G.; Yao Q.; Lin Y.; Gong P. Cyclic Mechanical Stress Modulates Neurotrophic and Myelinating Gene Expression of Schwann Cells. Cell Proliferation 2015, 48, 59–66. 10.1111/cpr.12151. 25418681 PMC6496414

[ref31] Martiáñez T.; Carrascal M.; Lamarca A.; Segura M.; Durany N.; Masgrau R.; Abian J.; Gella A. UTP Affects the Schwannoma Cell Line Proteome through P2Y Receptors Leading to Cytoskeletal Reorganisation. Proteomics 2012, 12, 145–156. 10.1002/pmic.201100187. 22065602

[ref32] Ruggeri M.; Bianchi E.; Rossi S.; Vigani B.; Bonferoni M. C.; Caramella C.; Sandri G.; Ferrari F. Nanotechnology-Based Medical Devices for the Treatment of Chronic Skin Lesions: From Research to the Clinic. Pharmaceutics 2020, 12, 815 10.3390/pharmaceutics12090815. 32867241 PMC7559814

[ref33] Rhim J. W.; Mohanty A. K.; Singh S. P.; Ng P. K. W. Effect of the Processing Methods on the Performance of Polylactide Films: Thermocompression versus Solvent Casting. J. Appl. Polym. Sci. 2006, 101, 3736–3742. 10.1002/app.23403.

[ref34] Esmaeilzadeh J.; Hesaraki S.; Hadavi S. M. M.; Esfandeh M.; Ebrahimzadeh M. H. Microstructure and Mechanical Properties of Biodegradable Poly (D/L) Lactic Acid/Polycaprolactone Blends Processed from the Solvent-Evaporation Technique. Mater. Sci. Eng.: C 2017, 71, 807–819. 10.1016/j.msec.2016.10.070. 27987776

[ref35] Régibeau N.; Tilkin R. G.; Compère P.; Heinrichs B.; Grandfils C. Preparation of PDLLA Based Nanocomposites with Modified Silica by in Situ Polymerization: Study of Molecular, Morphological, and Mechanical Properties. Mater. Today Commun. 2020, 25, 101610 10.1016/j.mtcomm.2020.101610.

[ref36] D’souza A. A.; Shegokar R. Polyethylene Glycol (PEG): A Versatile Polymer for Pharmaceutical Applications. Expert Opin. Drug Delivery 2016, 13, 1257–1275. 10.1080/17425247.2016.1182485. 27116988

[ref37] Ma Y.; Gao H.; Wang H.; Cao X. Engineering Topography: Effects on Nerve Cell Behaviors and Applications in Peripheral Nerve Repair. J. Mater. Chem. B 2021, 9, 6310–6325. 10.1039/d1tb00782c. 34302164

[ref38] Kijeńska E.; Prabhakaran M. P.; Swieszkowski W.; Kurzydlowski K. J.; Ramakrishna S. Electrospun Bio-Composite P(LLA-CL)/Collagen I/Collagen III Scaffolds for Nerve Tissue Engineering. J. Biomed. Mater. Res., B 2012, 100, 1093–1102. 10.1002/jbm.b.32676. 22438340

[ref39] Shah M. B.; Chang W.; Zhou G.; Glavy J. S.; Cattabiani T. M.; Yu X. Novel Spiral Structured Nerve Guidance Conduits with Multichannels and Inner Longitudinally Aligned Nanofibers for Peripheral Nerve Regeneration. J. Biomed. Mater. Res., Part B 2019, 107, 1410–1419. 10.1002/jbm.b.34233. PMC6438778 30265781

[ref40] Mohammadi M.; SaadatAbadi A. R.; Mashayekhan S.; Sanaei R. Conductive Multichannel PCL/Gelatin Conduit with Tunable Mechanical and Structural Properties for Peripheral Nerve Regeneration. J. Appl. Polym. Sci. 2020, 137, 49219 10.1002/app.49219.

[ref41] Gong B.; Zhang X.; Al Zahrani A.; Gao W.; Ma G.; Zhang L.; Xue J. Neural Tissue Engineering: From Bioactive Scaffolds and in Situ Monitoring to Regeneration. Exploration 2022, 2, 20210035 10.1002/exp.20210035. PMC10190951 37323703

[ref42] Ao Q.; Fung C. K.; Tsui A. Y.-P.; Cai S.; Zuo H. C.; Chan Y. S.; Shum D. K.-Y. The Regeneration of Transected Sciatic Nerves of Adult Rats Using Chitosan Nerve Conduits Seeded with Bone Marrow Stromal Cell-Derived Schwann Cells. Biomaterials 2011, 32, 787–796. 10.1016/j.biomaterials.2010.09.046. 20950852

[ref43] Liu K.; Yan L.; Li R.; Song Z.; Ding J.; Liu B.; Chen X. 3D Printed Personalized Nerve Guide Conduits for Precision Repair of Peripheral Nerve Defects. Adv. Sci. 2022, 9, 2103875 10.1002/advs.202103875. PMC9036027 35182046

[ref44] Geuna S.; Raimondo S.; Fregnan F.; Haastert-Talini K.; Grothe C. In Vitro Models for Peripheral Nerve Regeneration. Eur. J. Neurosci. 2016, 43, 287–296. 10.1111/ejn.13054. 26309051

[ref45] Junka R.; Yu X. Novel Acellular Scaffold Made from Decellularized Schwann Cell Sheets for Peripheral Nerve Regeneration. Regener. Eng. Transl. Med. 2015, 1, 22–31. 10.1007/s40883-015-0003-2. PMC4734393 26848489

[ref46] Junqueira L. C. U.; Cossermelli W.; Brentani R. Differential Staining of Collagens Type I, II and III by Sirius Red and Polarization Microscopy. Arch. Histol. Jpn. 1978, 41, 267–274. 10.1679/aohc1950.41.267. 82432

[ref47] Lattouf R.; Younes R.; Lutomski D.; Naaman N.; Godeau G.; Senni K.; Changotade S. Picrosirius Red Staining: A Useful Tool to Appraise Collagen Networks in Normal and Pathological Tissues. J. Histochem. Cytochem. 2014, 62, 751–758. 10.1369/0022155414545787. 25023614

